# Patient understanding of discharge instructions in the emergency department: do different patients need different approaches?

**DOI:** 10.1186/s12245-018-0164-0

**Published:** 2018-02-08

**Authors:** Hasan Sheikh, Aleksandar Brezar, Agata Dzwonek, Lawrence Yau, Lisa A. Calder

**Affiliations:** 1 Department of Emergency Medicine, University Health Network, Toronto, Ontario Canada; 2 Faculty of Medicine, University of Ottawa, Ottawa, Ontario Canada; 3 Ottawa Hospital Research Institute, Ottawa, Ontario Canada; 4 Department of Emergency Medicine, University of Ottawa, Ottawa, Ontario Canada

## Abstract

**Background:**

Previous studies have demonstrated that patients have poor understanding of the discharge instructions provided from the emergency department (ED). The aims of this study are to determine if patient factors, such as income and level of education, correlate with patient understanding of discharge instructions and to explore if different patient populations prefer different resources for receiving discharge instructions.

**Methods:**

We conducted live observations of physicians providing discharge instructions in the ED to 100 patients followed by a patient survey to determine their understanding in four domains (diagnosis, treatment plan, follow-up instructions, and return to ED (RTED) instructions) and collect patient demographics. We enrolled patients over the age of 18 being discharged home. We excluded non-English- or French-speaking patients and those with significant psychiatric history or cognitive impairment. We performed a two-way ANOVA analysis of patient factors and patient understanding.

**Results:**

We found that patients had poor understanding of discharge instructions, ranging from 24.0% having poor understanding of their follow-up plan to 64.0% for RTED instructions. Almost half (42%) of patients did not receive complete discharge instructions. Lower income was correlated with a significant decrease in patient understanding of discharge diagnosis (*p* = 0.01) and RTED instructions (*p* = 0.04). Patients who did not complete high school trended towards lower levels of understanding of their diagnosis and treatment plan (*p* = 0.06). Lower income patients had a preference for receiving a follow-up phone call by a nurse, while higher income patients preferred online resources.

**Conclusions:**

Lower income patients and those who have not completed high school are at a higher risk of poor understanding discharge instructions. As new technological solutions emerge to aid patient understanding of discharge instructions, our study suggests they may not aid those who are at the highest risk of failing to understand their instructions.

**Electronic supplementary material:**

The online version of this article (10.1186/s12245-018-0164-0) contains supplementary material, which is available to authorized users.

## Background

Health literacy in Canada is defined as “the ability to access, understand, evaluate and communicate information as a way to promote, maintain and improve health in a variety of settings across the life-course” [1]. More than half (55%) of Canadians are estimated to have less than adequate health literacy skills [1]. Higher health literacy scores have been correlated with higher self-reported health status [1], while lower literacy scores have been correlated with negative health outcomes, greater health care utilization, and increased costs [1–4]. Health care literacy is also related to the social determinants of health, including level of education and income [1, 5]. Importantly, the relationship between the social determinants of health and health outcomes can be attenuated by improvements in health literacy [6–9].

In the emergency department (ED), multiple studies have demonstrated that patients have poor understanding of the discharge instructions provided [3, 10–13]. Multiple interventions have been shown to improve patient understanding; however, many of the interventions discussed in the literature involve the increased use of technology, including online videos, email, and text messaging [14–17]. Given the relationship between low health literacy and the social determinants of health, lower income patients may be at the highest risk of poor understanding of their instructions and may be less able to access the interventions discussed in the literature. This study aims to understand how different socioeconomic groups understand discharge instructions and their preferences of how to receive discharge instructions. Our hope is that this will aid us in clarifying the current challenges our patients face in understanding discharge instructions and propose solutions to address this gap in understanding. Our primary objective was to explore the relationship between the social determinants of health (for example, level of education and income) and other characteristics (for example, being discharged in the last 1 h of the shift) and patient understanding of discharge instructions. Our secondary objective was to explore if different patient populations have different resource preferences for discharge instructions.

## Methods

### Study design and setting

We conducted a dual-phase study which included live observations of discharge instructions and a subsequent in person patient survey at The Ottawa Hospital from November 2014 to June 2015. The Ottawa Hospital is a large tertiary care academic hospital with dual campus adult emergency departments in Ottawa, Canada. Approximately 150,000 patients visit the emergency departments each year.

### Study population

We recruited a convenience sample of 100 patients who were discharged from the urgent care (lower acuity) area of the emergency department at either campus. We selected patients who were waiting for test results or treatment in the urgent care area of the emergency department in order to not affect the flow of the department. We excluded patients who were less than 18 years old, had significant psychiatric history, had significant dementia or cognitive impairment; patients who were not responsible for their own care at home; and non-English-speaking patients (English as a second language was included).

### Data collection

We planned data collection shifts in order to ensure a broad representation of times of day. We informed physicians working during those shifts of the study ahead of time by email and provided a verbal reminder to staff working in the emergency department on the data collection shift. The treating physician briefly introduced the study and obtained verbal consent from the patient to be informed about the study. If the patient agreed, one of three medical students will approach the patients and obtain informed written consent. We informed patients that data would be kept confidential and de-identified given the sensitivity of the data being collected.

When the physician was ready to discharge the patient, the medical student then observed the interaction and documented the instructions given by the discharging physician. Specifically, instructions were categorized into one of four predefined domains: diagnosis, treatment plan, follow-up plan, and instructions on when to return to the emergency department.

Immediately after the physician completed the discharge instructions, the student conducted a brief, standardized, in-person survey with the patient after the physician left the room. The student then collected additional information from the patient’s health record, including whether the patient was discharged in the last hour of the physician’s shift and the CTAS (Canadian Triage and Acuity Scale) score.

### Survey tool

We searched the literature and did not find a standardized survey tool to assess patient understanding of ED discharge instructions. Subsequently, we developed a survey tool and piloted it in the emergency department with 10 patients, modifying the questions according to feedback received by patients. Data from the piloted survey was not included in the analysis. The survey tool had a total of 13 questions, using a combination of open and more focused questions regarding the four domains in an attempt to not prompt patients (see Additional file 1). For example, in the treatment domain, patients were asked “Were you prescribed any medications today? If so, what medications were prescribed to you? Were you given any special instructions about the medications prescribed? If so, what were those instructions?” Patient characteristics were collected, including demographics, level of education, income, and what resources the patient believed would be the most helpful for them to understand their discharge instructions. The categories for level of education included not having completed high school, completed high school or equivalent, completed college or trade school equivalent, enrolled in or completed university, or other. The categories for annual household income were created based on Canadian Census data from 2011 and were split into three categories: < $25,000 (lowest quintile), $25,000–$90,000 (middle three quintiles), and > $90,000 (highest quintile). The categories for the preferred method of receiving discharge instructions included face-to-face discussion with the doctor, face-to-face discussion with the nurse, instruction sheet or handout, being directed to online resources, receiving a follow-up phone call by a nurse, watching a brief 3-min video in the ED, or other. Patients were allowed to select up to a maximum of two resources. Please refer to Additional file 1 for the complete survey tool.

### Outcome measures

Our primary outcome was to evaluate patient understanding of discharge instructions in the four domains. Based on previous research [3, 10, 13] quantifying patient understanding of discharge instructions, we evaluated patient understanding on a 4-point scale. The scale ratings included no, poor, adequate, and excellent understanding. Two physician reviewers blindly evaluated patient understanding using pre-specified rubric (see Additional file 2); any disagreements were resolved by consensus. In general, no understanding was defined as a lack of relationship between what the patient was told and what they reported, poor understanding was defined as a significant misunderstanding that could potentially lead to harm, adequate understanding was defined as a misunderstanding that was not expected to result in harm, and excellent understanding was defined as having no gaps in knowledge. Secondary outcomes were the correlation between patient characteristics (annual income, level of education, etc.) and degree of understanding and the correlation between patient characteristics and resource preference.

### Analysis

We described the study population and level of understanding using descriptive statistics. Each domain was analyzed separately using a two-way ANOVA analysis to assess for a relationship between different patients’ characteristics and level of understanding. We used a Mantel-Haenszel chi-square test to test for statistical significance. Patients who did not receive instructions in a particular domain were excluded from the analysis of that particular domain.

## Results

We approached 103 patients, with three refusals and 100 patients participating in the study. The mean age was 46 years old (with a standard deviation of 20), with 51.0% female respondents. In our sample, 39.0% of the patients had a household income of less than $25,000 a year, representing the lowest quintile of the population. Less than half (49.0%) of patients interviewed earned between $25,000 and $90,000, representing the middle three quintiles of income, while 19.0% of patients made over $90,000 a year. A small number of patients [2] elected not to provide data about their annual income. Just over one quarter (26.0%) of patients reported having difficulty making ends meet at the end of the month. A minority of patients (9.0%) did not complete high school. See Table 1 for the full description of the baseline characteristics of the study population. Nearly one quarter of the patients (23%) were discharged in the last 1 h of the shift; that 1 h represents one eighth (12.5%) of the typical 8 h emergency physician shift at The Ottawa Hospital.

**Table 1:** Baseline characteristics of the 100 study patients

Characteristics	Number
Age (mean in years, ±SD)	46 ± 20
Female	51
General campus	78
Average household income	
• < $25,000	39
• $25,000–90,000	49
• > $90,000	19
• Did not answer	2
Difficulty making ends meet at the end of the month	26
Level of education	
• Did not complete high school	9
• Completed high school or equivalent	31
• College education or trade diploma	25
• University education	35
• Patients with English/French not as their first language	27
CTAS	
• 1	0
• 2	22
• 3	57
• 4	19
• 5	2
Discharged in last 1 h of shift	23
Time of discharge	
• Day: 0800–1600	30
• Evening: 1600–2400	63
• Night: 2400–0800	7
Chief complaint	
• Abdominal/flank pain	12
• Back pain	3
• Chest pain/shortness of breath	11
• Extremity injury/pain	14
• Laceration/puncture	4
• Urinary complaints	8
• Others	48

Table 2 shows a more detailed description of patients’ understanding of their diagnosis, treatment, follow-up, and return to the emergency department instructions. Almost one third (28.6%) of patients had no or poor understanding of their diagnosis. Roughly one third (32.5%) of patients had no or poor understanding of their treatment plan. Finally, nearly one quarter (24.4%) of patients had no or poor understanding of their follow-up plan. Overall, the least understood domain was the return to ED instructions, with 63.8% of patients having no or poor understanding of when to come back to the ED to be reassessed.

**Table 2:** Percentage of patients with incomplete or no/poor understanding of discharge instructions in the four domains, of a total of 100 patients

	Incomplete understanding	No understanding or poor understanding
Diagnosis (*N* = 98)	47%	29%
Treatment (*N* = 86)	70%	33%
Follow-up (*N* = 86)	55%	24%
Return to ED (*N* = 80)	91%	64%

Note: Patients not provided with instructions for a particular domain were excluded from the analysis

Many patients did not receive complete discharge instructions. Table 3 shows a description of how many patients failed to receive instructions in a given domain. Almost half (42.0%) of patients did not receive instructions in at least one domain, with the most common area missed being the return to ED instructions (20.0%).

**Table 3:** Percentage of patients who did not receive discharge instructions in the various domains, of a total of 100 patients

Domain	Discharge instructions not provided
Diagnosis	2%
Treatment	14%
Follow-up	14%
Return to ED	20%
At least one domain	42%

Table 4 shows a univariate analysis examining the relationship between those patients with no or poor understanding and specific patient characteristics. Lower household income was correlated with a statistically significant decrease in patient understanding of their discharge diagnosis (*p* = 0.01) and return to ED instructions (*p* = 0.04). Level of education did not have a statistically significant relationship with level of understanding; however, patients who did not complete high school did have a trend towards less understanding of their discharge diagnosis (*p* = 0.06) and treatment plan (*p* = 0.06). Being discharged in the last 1 h of the shift did not have a significant impact on patient understanding of discharge instructions, or on whether the patient received complete instructions. Patients who stated that they had difficulty making ends meet at the end of the month and patients who were not primarily English or French speakers did not have a significant difference in their understanding of ED discharge instructions either.

**Table 4:** Percentage of patients (*N* = 100) with no or poor understanding of discharge instructions in the various domains of understanding, stratified by patient characteristics

		Discharge instruction domain							
Patient characteristic		Diagnosis		Treatment		Follow-up		RTED	
Income	< $25 k	44.7%		30.3%		32.4%		73.3%	
	$25–90 k	20.5%	*p = 0.01*	28.6%	*p* = 0.44	24.2%	*p* = 0.11	65.6%	*p = 0.04*
	> $90 k	15.8%		43.8%		11.8%		41.2%	
Level of education	No high school	55.6%		62.5%		37.5%		60.0%	
	Completed high school	25.8%	*p* = 0.06	29.5%	*p* = 0.06	23.1%	*p* = 0.37	64.0%	*p* = 0.86
Last 1 h of shift?	Yes	31.8%		21.1%		15.0%		72.2%	
	No	27.6%	*p* = 0.70	35.8%	*p* = 0.23	27.3%	*p* = 0.27	61.3%	*p* = 0.40
Difficulty making ends meet?	Yes	34.6%		29.2%		36.4%		66.7%	
	No	26.4%	*p* = 0.43	33.9%	*p* = 0.68	20.3%	*p* = 0.13	62.7%	*p* = 0.75

Note: italicized p values indicate statistically significant *p* values < 0.05

Table 5 shows the patients’ preferences for how they wished to receive discharge instructions, stratified by level of income. The majority of patients (81.0%) of all income levels felt that a face-to-face conversation with the physician was preferable. Only patients in the lowest income bracket expressed an interest in a follow-up phone call by a nurse (20.5% compared to 0.0% in the other income levels). Patients in the highest income bracket expressed an interest in online resources at a higher proportion than other patients (26.3% for those with a household income greater than $90,000 compared to 7.6% for others). For lower income patients, the two most preferred resources were face-to-face discussion with the physician and a physical handout. For higher income patients, the two most preferred resources were face-to-face discussion with the physician and face-to-face discussion with the nurse.

**Table 5:** Percentage of patients’ preference for different methods of receiving discharge instructions for the 100 patients surveyed. Patients were asked to provide a maximum of two methods

Preferred method for receiving discharge instructions	< $25,000 (%)	$25,000–$90,000 (%)	> $90,000 (%)	All patients
Face-to-face with MD	76.9	92.5	73.7	81
Face-to-face with RN	17.9	25	31.6	23
Follow-up phone call with RN	20.5	0	0	8
Physical handout	25.6	15	26.3	21
Online resources	7.7	7.5	26.3	11
Brief video in ED	2.6	2.5	0	2
None	2.6	2.5	5.3	3
Other	2.6	2.5	0	2

## Discussion

This study builds on existing research regarding patient understanding of discharge instructions in the emergency department. Our findings were consistent with previous studies that found that patients have a poor understanding of discharge instructions overall [10, 11, 13], with return to ED instructions being the least understood. In addition, a large proportion (42%) of patients did not receive instructions in one of the four domains. To our knowledge, this is the first study to look at what factors might help identify a subgroup of patients at higher risk of failing to understand ED discharge instructions. Our study suggests that lower income and not completing high school are predictors of lower levels of understanding. This is consistent with previous research that has shown that lower level of education is associated with lower health literacy, with the largest gap being between those that completed high school and those that did not [1]. Despite our findings regarding income and patient understanding, asking patients if they are having difficulty making ends meet did not correlate with patient understanding. This question has previously been used to screen for poverty in the outpatient setting [18], but our results did not find it discriminatory in identifying patients at risk of poor understanding. Our small sample size makes it difficult to generalize these results, and further research is necessary to determine the best way to identify patients at high risk of poor understanding of discharge instructions.

Our study is also the first to ask patients what resource they preferred for receiving discharge instructions, stratified by the social demographic factors that we collected. The most frequently preferred resource for the majority of patients was the face-to-face interaction with the treating physician; however, lower income patients had a preference for lower technology solutions such as a follow-up phone call with a nurse, while higher income patients had a preference for higher technology solutions such as online resources. Many of the studies evaluating interventions to improve patients’ understanding of discharge instructions rely on increased use of technology, for example, online videos and text messaging [14–17]. Our study suggests that while these interventions may be helpful in improving understanding, they may not aid the patients who are at the highest risk of failing to understand their instructions.

### Limitations

There are several limitations of this study. This was a non-random sample, and therefore, selection bias may have been introduced as the treating physician recruited the patients. Potentially, patients who the physician deemed less likely to understand the instructions may have been overlooked. In addition, the Hawthorne effect may have been a factor in this study, as both the patient and the physician were aware of the observers during the discharge instruction process. This may have led to a more complete set of instructions being given; however, it is also possible that it may have led to excessive instructions that could have overwhelmed patients and made it harder to recall the pertinent details. Additionally, medical students observed and recorded the discharge instructions and were not qualified to provide feedback to the treating physician. We also had a small sample size, and the study was conducted at a single urban tertiary care center, which may limit generalizability. Some groups of patients, for example, those that did not complete high school, were represented in small numbers, and therefore, conclusions related to this population should be cautiously drawn. The survey was administered immediately after the instructions were given, and it is unclear if immediate recall is correlated to long-term understanding of instructions when the patient arrives at home. Instructions may also have been given during the initial history and physical exam and may not have been repeated at the time of discharge, which would be interpreted as a failure to provide complete instructions in this study. Finally, we did not assess the quality of the discharge instructions themselves.

### Clinical and research implications

Our data demonstrate that a significant number of patients have no or poor understanding of their discharge instructions, and many patients fail to receive complete discharge instructions. We identified a group of patients that are at high risk for not understanding discharge instructions, including low income patients and those who did not complete high school. Further research will be needed to find the best way to identify these patients in real time at the bedside. In a busy emergency department, however, these results may prompt clinicians to take extra time with patients from lower socioeconomic backgrounds to ensure that they have understood their instructions. Our study suggests that lower income patients may have a different preference for resources for aiding understanding of discharge instructions, and further research is needed to confirm these findings, explore the reasons behind their preferences, and determine whether interventions addressing these needs mitigate the difference in understanding between low- and high-income patients.

## Conclusions

This observational study confirmed previous research that patient understanding of discharge instructions in the ED is poor. In addition, lower income patients and those who have not completed high school are at a higher risk of failing to understanding discharge instructions. Our data suggest that patients of different socioeconomic backgrounds may have different preferences for resources to aid their understanding. Clinicians should be aware of this potential difference and consider a follow-up phone call to low-income patients and those who have not completed high school, in order to ensure that they have understood their instructions. As we continue to explore potential solutions to aid patient understanding, especially technologically focused ones, we should be cognizant that they may not aid the patients who are at the highest risk of failing to understand their instructions.

## Additional files

Additional file 1: Discharge instructions from the emergency department: Investigating a customized. (PDF 75 kb)

Additional file 2: Supplementary material. (PDF 43 kb)
