# Supplementary material for: Patient understanding of discharge instructions in the emergency department: do different patients need different approaches?
Source: Int J Emerg Med. 2018 Feb 8;11:5. doi: 10.1186/s12245-018-0164-0 (PMC5805670; doi:10.1186/s12245-018-0164-0)
Supplement: Supplementary file 1 — Discharge instructions from the emergency department: Investigating a customized. (PDF 75 kb) [file 12245_2018_164_MOESM1_ESM.pdf]

Discharge Instructions from the Emergency Department: Investigating a Customized Approach

Data Collection Form: Version 2, April 16, 2015

Patient interview

Study Number: ___________________

1. What is your native language?
   A. English
   B. French
   C. Other: __________________
2. What diagnosis were you given today in the Emergency Department?

   ____

3. Were you told about a follow up plan after your ED visit?
   A. Yes/No
   B. If so, what was the follow-up plan?

   ____

   ____

4. Were you prescribed any medications today?
   A. Yes/No
   B. If so, what medications were prescribed to you?

   ____

   ____

5. Were any special instructions given to you about the medications prescribed?
   A. Yes/No

B. If so, what were the special instructions?

____

____

6. Were you told about other treatments, including over the counter medications?

   A. Yes/No

   B. If so, what treatments were you told about?

____

____

7. Were any special instructions given to you about that treatment?

   A. Yes/No

   B. If so, what were the special instructions?

____

____

8. Were you told of reasons that you should return to the Emergency Department?

   A. Yes/No

   B. If so, what were the reasons you should return to the Emergency Department?

____

____

9. What resource would be the most helpful for you to understand today's diagnosis and follow up instructions?

   A) Please pick a maximum of two.

      A. Face-to-face discussion with Doctor

      B. Face-to-face discussion with Nurse

      C. Instruction Sheet or Handout

D. Being directed to online resources

E. Follow up phone call by a Nurse

F. Brief 3 minute video in ED

G. Other: ________________

B) What language would you like those resources in?

____

10. What level of education have you obtained?

A. Did not complete High School or equivalent

B. Completed High School or equivalent

C. College education or Trade equivalent

D. University education - completed or enrolled

E. Other: ______________________________

11. Which annual household income bracket applies to you?

A. < $25,000

B. $25,000 - $90,000

C. > $90,000

12. Do you ever have difficulty making ends meet at the end of the month?

A. Yes

B. No

13. Do you have any comments regarding the instructions you received today?

Thank you for your participation in this study. Your help is greatly appreciated.

Discharge Instructions from the Emergency Department: Investigating a Customized Approach

Data Collection Form: Version 1, March 23 2015

ED visit characteristics

Study Number: ______________

1. Age: ________________
2. Sex:
   A. Male
   B. Female
3. Presenting complaint:

   ____
4. CTAS:
   A. 1
   B. 2
   C. 3
   D. 4
   E. 5
5. Patient triaged to:
   A. Urgent
   B. Observation
   C. Emergent
   D. Resuscitation
6. Handover patient from a previous physician?
   A. Yes

B. No

7. Patient evaluated by (select all that apply):

    A. Staff Emergency Physician

    B. Emergency Medicine Resident

    C. Off-service Resident

    D. Medical Student

8. Discharge instructions provided by:

    A. Staff Emergency Physician

    B. Emergency Medicine Resident

    C. Off-service Resident

    D. Medical Student

9. Time of discharge:

    Actual time of discharge: ________________

    A. 0800-1600

    B. 1600-2400

    C. 0000-0800

Discharge Instructions from the Emergency Department: Investigating a Customized Approach

Data Collection Form: Version 2, April 16, 2015

Observation record of discharge instructions provided by physician

Study Number: _______________

1. Discharge Diagnosis:

    ____

    ____

2. Follow up instructions:

    ____

    ____

3. Medications prescribed:

    ____

    ____

4. Special instructions for taking prescribed medications

    ____

    ____

5. Other treatments recommended, including over the counter medications

    ____

    ____

6. Special instructions for other treatments

    ____

    ____

7. Return to the Emergency Department if:

____

____
