# Supplementary material for: Patient understanding of discharge instructions in the emergency department: do different patients need different approaches?
Source: Int J Emerg Med. 2018 Feb 8;11:5. doi: 10.1186/s12245-018-0164-0 (PMC5805670; doi:10.1186/s12245-018-0164-0)
Supplement: Supplementary file 2 — Supplementary material. (PDF 43 kb) [file 12245_2018_164_MOESM2_ESM.pdf]

	No understanding	Poor understanding	Adequate understanding	Excellent understanding
**Diagnosis**	Understood diagnosis has no relation to given diagnosis	Significant difference	Understood diagnosis not dangerous or harmful	Understood diagnosis (nearly) identical to given diagnosis
**Follow up**	No understanding of follow up plan	Significant misunderstanding, could result in poor care	Not complete understanding but no expected harm	Understands all components of follow up plan
**Treatment**	No relation	Significant misunderstanding that could result in harm	Not complete understanding but no expected harm	No gaps in knowledge of treatment including special instructions
**RTER**	Does not know about RTER instructions	Significant misunderstanding that could result in harm	Not complete understanding but no expected harm	No gaps in knowledge
